# Effects of dried *Azolla pinnata* supplementation on growth, biochemistry, and reproduction in female rabbits

**DOI:** 10.1007/s11250-026-04966-2

**Published:** 2026-03-31

**Authors:** Omnia Y. Abd-Elfadiel Hagag, Muhammed Ahmed-Helmy El-Rayes, Ahmed S. El-Hawy, Zhou I. Nabil, Nahla Soliman El-Shenawy

**Affiliations:** 1 Department of Zoology, Faculty of Science, Suez Canal University, Ismailia, 4153 Egypt; 2 Animal and Poultry Physiology Department, Desert Research Center, Cairo, Egypt

**Keywords:** *Azolla pinnata*, New Zealand white rabbits, Growth performance, Productivity, Soybean meal protein, Biochemical parameters, Immunity, Hormones

## Abstract

This study evaluated the impact of incorporating varying levels of dried *Azolla pinnata* as a partial or full substitute for soybean meal protein on growth performance, productivity, and selected biochemical, immunological, hormonal, and antioxidant parameters in New Zealand White (NZW) female rabbits. The study conducted at the South Sinai Research Station of the Desert Research Center, Egypt, from May to November 2023. The trial involved 45 eight-week-old NZW female rabbits. These were randomly assigned to three dietary groups: a control group fed a standard concentrate diet, a group where 50% of soybean protein was replaced with Azolla (GΙ), and a group where soybean protein was completely replaced with Azolla (GΠ). Findings revealed that the GΙ group (50% Azolla replacement) achieved superior growth performance throughout the study period. Conversely, the GΠ group (100% Azolla replacement) experienced a significant reduction in body weight during later growth stages. Regarding productivity, no marked differences among the groups were observed in birth numbers. However, birth mortality rates were notably lower in the GΠ group, despite a significant reduction in kit weights during the first four weeks postpartum. Biochemical analyses showed that Azolla inclusion affected blood parameters based on supplementation level and duration. In the first month, the GΠ group exhibited elevated total protein and globulin concentrations alongside a reduced albumin-to-globulin ratio, while the trend reversed in the second month. During pregnancy, total protein and globulin levels increased in both supplemented groups. Additionally, Azolla influenced kidney and liver function markers, lipid profiles, immunoglobulin concentrations, thyroid hormones, and reproductive hormones, with variations noted across groups and stages of the experiment. Therefore, partial replacement of soybean meal protein with 50% dried *A. pinnata* improved growth performance without adverse effects on reproductive outcomes or biochemical balance in NZW female rabbits. However, complete replacement (100%) negatively impacted growth and certain productivity parameters, indicating that while Azolla has beneficial properties, its optimal use lies in partial substitution rather than total replacement to maintain rabbit health and performance.

## Introduction

The world is witnessing a steady and unprecedented increase in food demand due to population growth, urbanization, and shifting dietary preferences (Alshelmani et al. 2021, 2024). Projections indicate that the global population will approach 9.7 billion by 2050, necessitating a 60% increase in agricultural output to ensure food security (van Dijk et al. 2021; FAO, 2023). Recent global events—most notably the COVID-19 pandemic and ongoing geopolitical instabilities—have exposed critical vulnerabilities in international food supply chains, renewing emphasis on resilient local food systems and alternative feed resources (Love et al. 2021).

Animal agriculture, particularly non-ruminant sectors such as rabbit production, is acutely affected by the scarcity and high costs of conventional feed ingredients. The escalating demand for animal products is driving up the use of staple crops like soybean, maize, barley, and wheat—and, consequently, contributing to increased competition between human and animal nutritional needs exacerbating concerns around environmental sustainability and economic feasibility (Ogbuewu et al. 2017; Abdelhadi et al. 2022). These challenges underscore the urgent need to identify and utilize locally available, low-cost, and sustainable alternative protein sources to mitigate feed costs and support long-term food system resilience (Abdelatty et al. 2021; El Naggar and El-Mesery 2022).

One of the most significant challenges in this context is securing easily accessible protein sources capable of meeting the nutritional requirements of non-ruminant animals at a low cost. Among potential alternatives, aquatic macrophytes like Azolla have shown promising outcomes (Méndez-Martínez et al. 2019). Several factors contribute to the unsustainability of current feed imports in some countries, where key ingredients such as soybean meal, fishmeal, and maize-based feed ingredients (e.g., corn grain, corn gluten meal, or corn gluten feed) are imported instead of being domestically produced (Nasir et al. 2022).

The widespread reach of media has increased public awareness regarding the importance of high-quality, complete protein foods derived from animals, characterized by high nutritional and biological value. This heightened awareness has subsequently boosted the demand for such foods (Anitha et al. 2016). The need for meat with high nutritional value, specifically low fat, high protein, and low sodium content, coupled with rapid human population expansion, is the primary driver behind the increased production of rabbit meat (Mottet and Tempio 2017).

Non-ruminant species such as rabbits are highly efficient in feed conversion and play an increasingly important role in providing high-quality, low-fat, and protein-rich meat with favorable lipid profiles (Siddiqui et al. 2024). However, traditional rabbit diets largely depend on crops like soybean, maize, and wheat—commodities that directly compete with human consumption and require intensive land and water resources (Odero-Waitituh et al. 2021). Consequently, the exploration of sustainable, locally available, and nutritionally adequate alternatives is essential for ensuring both food security and economic viability.

Rabbit and poultry diets often include ingredients from key crops like soybean, corn, barley, and wheat—staples also used in human food (Ogbuewu et al. 2017). To avoid competition with human consumption and keep rabbit meat production sustainable, it’s important to find alternative protein sources for their feed (Abdelatty et al. 2021).

Aquatic macrophytes such as *Azolla pinnata* have emerged as promising alternative feed ingredients. Azolla is a free-floating aquatic fern that forms a symbiotic association with nitrogen-fixing cyanobacteria (*Anabaena azollae*), resulting in high protein content (13–30%), beneficial amino acid profiles, and significant mineral and vitamin levels (Rashad et al. 2021). Its ease of cultivation, high yield with minimal water requirement, and digestibility further add to its appeal as a sustainable feed component (Kollah et al. 2016; Korsa et al. 2024). Incorporation of dried *A. pinnata* in rabbit diets has been shown to enhance production performance, body weight gain, and meat quality, with no negative impacts on feed intake, health, or reproductive performance (El-Sabrout et al. 2023; Nayel et al. 2024).

Azolla, a free-floating water fern, is rich in protein and essential amino acids, growth-promoting intermediates, vitamins (A, B12, and beta-carotene), and minerals such as calcium, phosphorus, potassium, iron, copper, and manganese. Furthermore, its high fiber content and low lignin level make it easily digestible for livestock. Azolla is considered one of the most promising alternatives due to its ease of cultivation, low water requirement for growth, high yield, and high nutritional content (Herath et al. 2023).

Recent studies demonstrate that sun-dried Azolla meal can partially or fully replace conventional protein sources such as soybean meal in rabbit diets, improving body weight gain, feed efficiency, and even meat quality, with no detrimental effects on health or reproductive outcomes (Nasir et al. 2022; Khan et al. 2024). Azolla’s ability to enhance performance, reduce environmental impact, and cut feed costs (Arora et al. 2022; Kouchakinejad et al. 2024; Kairalla et al. 2025). However, data on its application in rabbit diets—particularly its impact on female reproductive performance, metabolic health, and systemic biochemical responses—remain limited and inconsistent.

Accordingly, the present study aimed to evaluate the effects of partially and fully replacing soybean meal protein with sun-dried *A. pinnata* on growth performance, reproductive traits, and selected biochemical, hormonal, immunological, antioxidant parameters and carcass characteristics in New Zealand White (NZW) female rabbits. The findings aim to advance sustainable feeding practices and promote resource-efficient rabbit production, particularly in climate-challenged regions.

## Materials and methods

The experiment was conducted at the South Sinai Research Station of the Desert Research Center, Ministry of Agriculture, Egypt, from May to November 2023. All procedures were conducted in accordance with the ethical guidelines approved by the Research Ethics Committee, Faculty of Science, Suez Canal University, Ismailia, Egypt (Approval No. REC175/2022).

### Azolla cultivation and meal production

*A. pinnata* was cultivated in six excavated ponds (dimensions: 3 × 4 × 0.3 m) within a greenhouse environment. Each pond was prepared by lining the base with fertile soil and fertilizing with a mixture of rabbit manure slurry and superphosphate. Approximately 5 kg of fresh Azolla culture was inoculated into each pond. After 10–15 days, daily harvests of approximately 10 kg were collected following the method described by Adzman et al. (2022) method. The harvested biomass was sun-dried and ground into a fine meal. The chemical composition of the dried *Azolla* meal was analyzed using standard methods described by AOAC (2005) (Table 1).

**Table 1:** Chemical analysis of dried Azolla

Nutrients	%
Dry matter	9.1
Organic matter	79.4
Crude protein	19.4
Ether extract	3.3
Crude ash	20.6
Carbohydrates	41.4
Crude fiber	15.3

### Experimental design

A total of 45 healthy, 8-week-old NZW female rabbits with an average initial body weight of 1.535 ± 0.025 kg were randomly assigned to one of three equal dietary treatment groups (15 rabbits per group). Each rabbit was considered an independent biological replicate, providing 15 replicates per treatment, which ensures adequate statistical power and reliability of the results.

- Control: Basal concentrate diet without Azolla.
- GΙ: Basal diet with 50% replacement of soybean meal protein with *A. pinnata.*
- GΠ: Basal diet with 100% replacement of soybean meal protein with *A. pinnata.*

Rabbits were housed individually in standard wire cages (50 × 50 × 35 cm) under identical environmental and management conditions. All animals received their respective diets and had free access to clean drinking water. Prior to the experimental period, a 7-day adaptation phase was conducted.

Diets were formulated based on the nutrient requirements recommended by the National Research Council (NRC, 1977), and feed composition is presented in Table 2. The diets were prepared at the Ras Sudr Research Station and analyzed for their proximate composition using AOAC (2005) methods.

**Table 2:** Feed ingredients and chemical analysis of the experimental diets

Ingredients %	Control	GΙ	GΠ
Alfalfa hay	29	29	29
Yellow corn	23	23	23
soybean	18	9	-
Azolla	-	9	18
Wheat bran	24	24	24
Molasses	1	1	1
Limestone	2	2	2
Ca3(PO4)2	1	1	1
Vit. and Min	0.5	0.5	0.5
mix**	0.5	0.5	0.5
Salt	0.5	0.5	0.5
Methionine	0.25	0.25	0.25
Lysine	0.25	0.25	0.25
Total	100	100	100
Chemical analysis *	**Control**	**GΙ**	**GΠ**
Dry matter DM%	92.50	92.59	93.27
Organic matter OM%	90.81	89.64	88.65
Crude protein CP%	20.10	15.65	12.22
Ether extract EE%	4.49	4.81	6.29
Ash%	9.19	10.36	11.36
Nitrogen-free extract NDF%	55.38	56.85	52.05
acid detergent fiber ADF%	18.93	22.35	22.66
NFC%	10.85	12.35	18.10
gross energy GE (MJ/Kg DM)	18.23	17.81	17.73

Each one kg of vitamin & mineral mixture contains: Vit.A 4,000,000 IU; Vit D3 50000IU; Vit E 16.7 g.; Vit K3,0.67 g.; Vit.B1 67 g; VitB2 2.00 g; Vit. B6 0.67 g; Vit B12 3.33 mg; Cholin chloride 400 g.; Biotin 0.07 g; Niacin 16.7 g.; pantothenic acid 6.7 g; Folic acid 1.7 g; Copper 1.7 g; Iron 25.00 g; Manganese 10.00 g; Iodine 0.25 g; Selenium 33.3 g; Zinc 23.3 g and Magnesium 133.3 g. * Calculated according to NRC (1977). GΙ and GΠ are basal diet with 50% and 100% replacement of soybean meal protein with *A. pinnata*, respectively

**Fig. 1:**
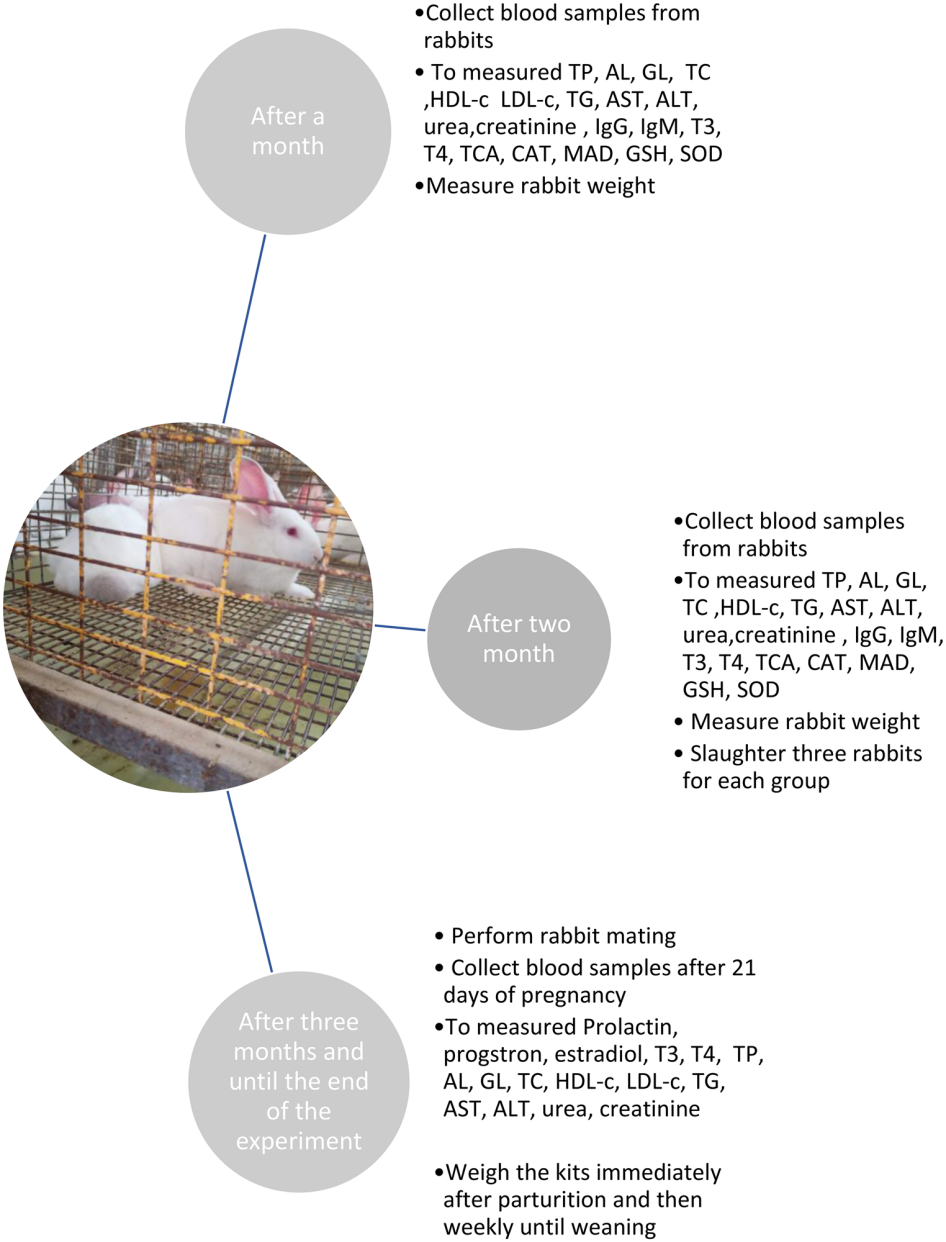
Diagram shows the experimental design

After 12 weeks of feeding, natural mating was conducted using a ratio of one buck to five does. Pregnancy was confirmed by abdominal palpation on day 15 post-mating (Fig. 1).

### Growth performance assessment

Individual body weights were recorded biweekly (before the morning feeding) throughout the 12-week feeding period. Kit weights were recorded immediately at birth and monitored weekly until weaning.

### Serum biochemical analysis

Blood samples were collected from the marginal auricular vein following an overnight fast at three points: after one month of feeding, after two months of feeding, and on day 21 of pregnancy. Samples were allowed to clot and then centrifuged at 3,000 rpm for 15 min. The separated serum was stored at − 20 °C for subsequent analysis.

The following biochemical parameters were evaluated using colorimetric assays and commercial diagnostic kits included total protein (Henry 1964), albumin (Doumas et al. 1971), calculated globulin, urea (Patton and Grouch 1977), creatinine (Bartel et al. 1972), alanine aminotransferase (ALT), aspartate aminotransferase (AST) (Reitman and Frankel 1957), triglycerides (Fassati and Prencipe 1982), total cholesterol (Allen 1974), and high-density lipoprotein cholesterol (HDL-C) (Lopez 1977). Low-density lipoprotein cholesterol (LDL-C) and very-low-density lipoprotein cholesterol (VLDL-C) were calculated using the formulas of Lee and Nieman (1996):

- VLDL-C = TG / 5.
- LDL-C = Total cholesterol – HDL-C – VLDL-C.

All assays were performed using commercially available kits, following the manufacturers’ instructions.

### Immunoglobulin and hormonal assays

Serum levels of immunoglobulin G (IgG) and immunoglobulin M (IgM) were measured using specific ELISA kits (Bio-Diagnostic, Egypt). Thyroid hormones—triiodothyronine (T3) and thyroxine (T4)—were assessed using radioimmunoassay (RIA) kits. Serum levels of prolactin and progesterone were quantified using enzyme-linked immunosorbent assay (ELISA) kits.

### Oxidative stress and antioxidant status evaluation

Lipid peroxidation was evaluated by measuring malondialdehyde (MDA) levels using a competitive binding ELISA method. Superoxide dismutase (SOD) activity was assessed using a rat SOD ELISA Kit (Cusabio, USA). Catalase (CAT) activity was determined using a double-sandwich ELISA method (MyBioSource, USA). Glutathione (GSH) levels were measured using an ELISA Kit (Cusabio, USA). Total antioxidant capacity (TAC) was assessed using the OxiSelectTM kit (Cell Biolabs, USA). All procedures followed the manufacturers’ instructions.

### Effect of Azolla on carcass traits of growing rabbits

After the two-month experimental period (at 16 weeks of age), nine rabbits—three randomly selected from each group—were fasted for 15 h prior to assessment. The weights of giblets (liver, kidneys, heart, and spleen) and carcass measurements were recorded and expressed as percentages of live body weight. The dressing percentage was calculated following the method described by Lukefahr et al. (1982).

### Statistical analysis

Mortality rate was recorded daily throughout the experimental period. As no mortality occurred in any of the experimental groups (0%), mortality data showed no variability and therefore did not meet the assumptions required for inferential statistical analysis. Consequently, mortality was reported descriptively and excluded from further statistical testing. No data transformation was required due to the absence of mortality events.

Results were expressed as mean values ± standard error (SE). Differences among dietary treatments were evaluated using one-way analysis of variance (ANOVA). When significant differences were detected, means were separated using Tukey’s honestly significant difference (HSD) post-hoc test. Statistical significance was set at *P* ≤ 0.05. All analyses were performed using Minitab software.

## Results

### Growth performance

**Fig. 2:**
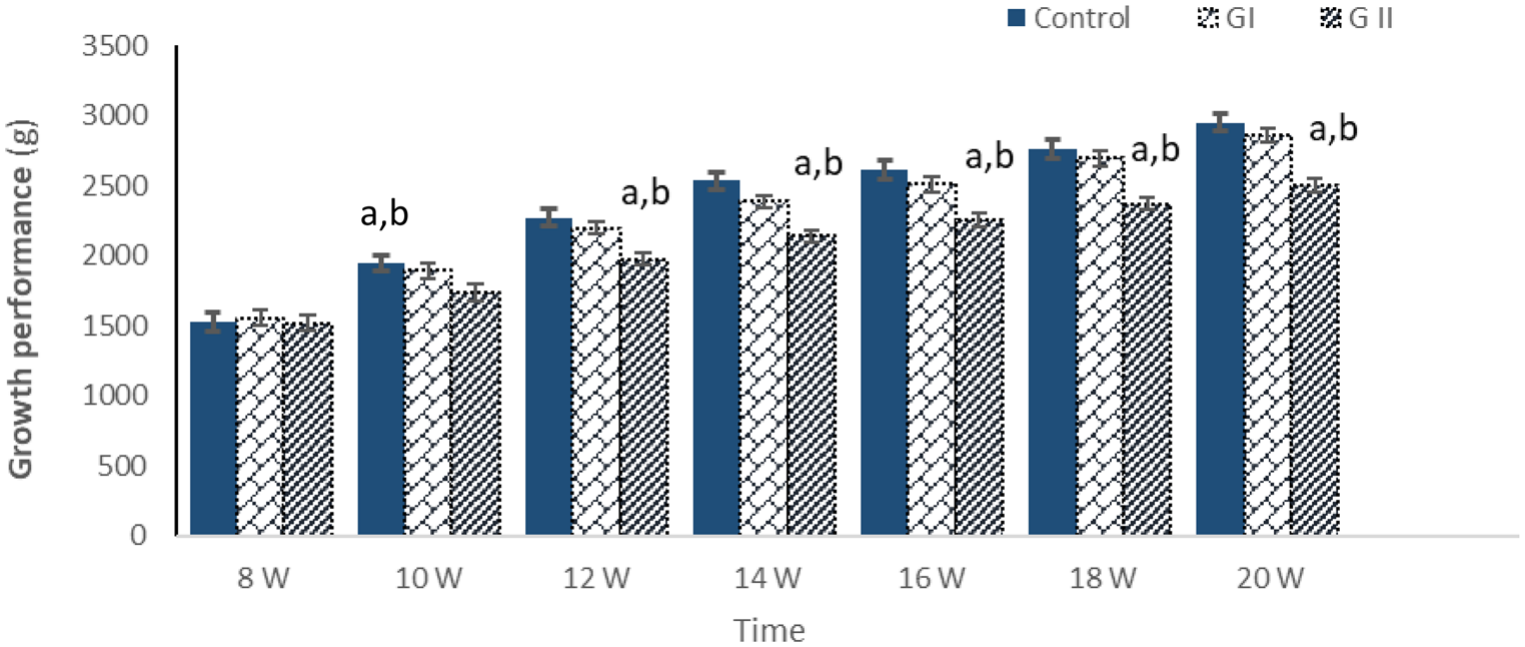
The effect of replacing soybean meal protein with dried Azolla on the growth performance of growing rabbits. Values presented as means ± SE (*n* = 15). GΙ; Azolla (50% of soybean protein), GΠ; Azolla (100% of soybean protein). ^a^ significant difference compared to control, and ^b^ significant differences compared to GΙ (*P* < 0.05)

Figure 2 illustrates the effect of partially or completely replacing soybean meal protein with dried *A. pinnata* on the weekly growth performance of young rabbits across the three dietary treatments. The control group (0% Azolla) showed a progressive increase in body weight from 1531.53 ± 60.54 g at week 8 to 2950.00 ± 62.67 g at week 20. Rabbits in the GI group (50% replacement) exhibited consistently higher body weights than those in the GII group (100% replacement) throughout the experimental period, increasing from 1556.66 ± 60.00 g at week 8 to 2860.66 ± 50.65 g at week 20. Body weights in the GI group were significantly higher than those of the GII group (*P* = 0.0003), indicating superior growth performance with partial replacement.

In contrast, the GII group recorded the lowest body weights at all measured time points. One-way ANOVA revealed significant differences among the three groups at several weeks (*P* < 0.05). From weeks 12 to 20, body weights in the GII group were markedly lower than those of both the control and GI groups (*P* < 0.001 at each time point). These findings demonstrate that complete replacement of soybean protein with *A. pinnata* adversely affects growth performance in growing rabbits.

Regarding reproductive performance, the number of births, birth weights, and overall mortality rates did not differ significantly among the experimental groups compared with the control (*P* > 0.05; Table 3). However, the GII group showed a significantly lower birth mortality rate compared with the control and GI groups (*P* = 0.032). In addition, kits from the GII group exhibited a significant reduction in body weight between the first and fourth weeks of age compared with both the GI and control groups (*P* = 0.018). A smaller but still significant decline in body weight over the same period was also observed in the GI group compared with the control (*P* = 0.041), as shown in Table 3.

**Table 3:** The effect of replacing soybean meal protein with dried Azolla on the weight, number, and mortality rate of births

Azolla (% of soybean protein)			
	Control	GΙ	GΠ
number of births	7.86 ± 0.35	8.33 ± 0.16	7.43 ± 0.29
mortality rate	3.29 ± 0.62	2.00 ± 0.44	1.71 ± 0.42^a, b^
Birth weight (g)	51.67 ± 1.88	53.00 ± 2.51	47.71 ± 1.41^a, b^
BW at 1 weeks (g)	116.67 ± 4.33	123.00 ± 8. 22	95.57 ± 2.70^a, b^
BW at 2 weeks (g)	219.83 ± 8.15	217.17 ± 10.02	187.71 ± 12.44^a, b^
BW at 3 weeks (g)	328.67 ± 10.38	323.50 ± 10.38	269.00 ± 14.20^a, b^
BW at 4 weeks (g)	460.67 ± 5.84	429.83 ± 9.35^a^	374.71 ± 13.57^a, b^

Values are presented as means ± SE (*n* = 7**).** GΙ and GΠ are basal diet with 50% and 100% replacement of soybean meal protein with *A. pinnata*., respectively ^a^ significant difference compared to control, and ^b^ significant differences compared to G Ι (*P* < 0.05)

### Effect of Azolla on serum biochemical parameters

Table 4 presents the effects of substituting soybean meal protein with dried *Azolla* in female rabbit diets on serum protein fractions, liver enzymes, lipid profile, and kidney function at different sampling intervals.

**Table 4:** Effect of soybean meal protein substitution with dried Azolla Serum Biochemical Parameters of growing rabbits at different times

After a month	Group	AST (U/l)	ALT (U/l)	TP (g/dL)	AL (g/dL)	GL (g/dL)	AL/GL (g/dL)	Urea (mg/dl)	Creatinine (mg/dl)	TC	HDL-c	LDL-c	TG	VLDL
	Control	19.37 ± 0.42	16.38 ± 0.73	5.04 ± 0.14	2.49 ± 0.11	2.64 ± 0.4	1.07 ± 0.04	39.62 ± 2.45	0.80 ± 0.04	49.83 ± 1.25	22.83 ± 0.79	19.10 ± 1.36	59.77 ± 2.34	11.95 ± 0.47
	G I	19.50 ± 0.59	17.45 ± 0.85	4.87 ± 0.10	2.40 ± 0.21	2.48 ± 0.23	0.97 ± 0.18	29.73 ± 0.72^a^	0.83 ± 0.03	57.77 ± 1.59^a^	27.22 ± 1.10^a^	18.10 ± 0.88	70.97 ± 1.97^a^	14.19 ± 0.39^a^
	G II	**10.37 ± 0.70** ^**a, b**^	**12.33 ± 0.62** ^**a, b**^	**5.83 ± 0.06** ^**a, b**^	**2.54 ± 0.09**	**3.29 ± 0.13** ^**a, b**^	**0.79 ± 0.60** ^**a, b**^	**35.87 ± 0.39** ^**b**^	**0.87 ± 0.07**	**79.40 ± 4.55** ^**a, b**^	**33.87 ± 2.15** ^**a, b**^	**26.20 ± 1.95** ^**a, b**^	**90.88 ± 3.26** ^**a, b**^	**18.18 ± 0.65** ^**a, b**^
**After two months**	**Control**	**17.70 ± 0.79**	**25.71 ± 0.70**	**6.10 ± 0.03**	**2.60 ± 0.14**	**3.50 ± 0.11**	**0.76 ± 0.06**	**47.5 ± 2.04**	**0.81 ± 0.04**	**48.38 ± 2.51**	**22.35±0.163**	**14.83 ± 1.01**	**57.63 ± 1.64**	**11.52 ± 0.32**
	**G I**	**21.11 ± 0.99** ^**a**^	**20.50 ± 1.31** ^**a**^	**6.00 ± 0.19**	**2.7 ± 0.07**	**3.30 ± 0.16**	**0.81 ± 0.38**	**68.16 ± 1.77** ^**a**^	**0.83 ± 0.03**	**62.53 ± 2.31** ^**a**^	**24.33 ± 10.8**	**28.10 ± 0.90** ^**a**^	**57.85 ± 3.11**	**11.93 ± 0.62**
	**G II**	**17.7 ± 1.88** ^**b**^	**22.65 ± 1.57**	**5.73 ± 0.10** ^**a, b**^	**2.61 ± 0.27**	**2.90 ± 0.05** ^**a, b**^	**0.97 ± 0.12** ^**a, b**^	**36.16 ± 0.74** ^**a, b**^	**0.77 ± 0.01**	**81.00 ± 3.74** ^**a, b**^	**44.50 ± 1.80** ^**a, b**^	**19.78 ± 1.40** ^**a, b**^	**72.70 ± 6.37** ^**a, b**^	**14.54 ± 1.27** ^**a, b**^
**After 21 days of pregnancy**	**Control**	**16.89 ± 0.93**	**15.06 ± 0.72**	**4.9 ± 0.05**	**2.67 ± 0.07**	**2.23 ± 0.08**	**1.20 ± 0.06**	**47.01 ± 0.93**	**0.68 ± 0.03**	**30.00 ± 1.82**	**14.25 ± 1.27**	**5.35 ± 0.3**	**42.81 ± 2.39**	**8.56 ± 0.47**
	**G I**	**23.61 ± 1.46** ^**a**^	**29.78 ± 0.73** ^**a**^	**6.03 ± 0.15** ^**a**^	**2.71 ± 0.09**	**3.31 ± 0.20** ^**a**^	**0.85 ± 0.08** ^**a**^	**39.67 ± 0.80** ^**a**^	**0.73 ± 0.03**	**50.08 ± 3.39** ^**a**^	**28.33 ± 1.54** ^**a**^	**11.90 ± 1.28** ^**a**^	**49.88 ± 3.64**	**9.97 ± 0.72**
	**G II**	**16.78 ± 0.62** ^**b**^	**17.55 ± 1.69** ^**b**^	**5.65 ± 0.16** ^**a**^	**2.5 ± 0.07**	**3.10 ± 0.23** ^**a**^	**0.81 ± 0.07** ^**a**^	**44.50 ± 2.59** ^**b**^	**0.72 ± 0.04**	**49.75 ± 3.78** ^**a**^	**24.20 ± 1.55** ^**a, b**^	**20.50 ± 1.94** ^**a, b**^	**64.16 ± 3.75** ^**a, b**^	**12.83 ± 0.75** ^**a, b**^

Values are presented as means ± SE (*n* = 7). G I; Azolla (50% of soybean protein), G II; Azolla (100% of soybean protein). ^a^ significant difference as compared to control, and ^b^ significant differences as compared to G I (*P* < 0.05). Aspartate aminotransferase (AST) and alanine aminotransferase (ALT). total protein (TP), albumen (AL), globulin (GL), and albumen/globulin ratio (A/G). Total cholesterol (TC), high-density lipoprotein (HDL-C), low-density lipoprotein (LDL-C), triglycerides (TG) and very low-density lipoprotein (VLDL-C)

After one month, rabbits in GII (100% replacement) exhibited significantly higher total protein (TP) and globulin (GL**)** levels compared with both the GI (50% replacement) and control groups (*P* = 0.002 and *P* = 0.001, respectively). Concurrently, the albumin: globulin (AL: GL) ratio was significantly reduced in GII relative to GI and the control (*P* < 0.001).

After two months, TP and GL levels in GII declined significantly compared with GI and the control (*P* = 0.004 and *P* = 0.006, respectively). In contrast, the AL: GL ratio increased markedly in GII relative to the other groups (*P* < 0.001). At 21 days of pregnancy, both substitution groups showed significantly higher TP and GL concentrations than the control (*P* < 0.05), whereas the AL: GL ratio remained significantly lower in GI and GII compared with the control (*P* < 0.01).

Regarding liver enzymes, GII showed significantly lower ALT and AST activities after one month compared with GI and the control (*P* = 0.008 and *P* = 0.006, respectively). However, after two months and at 21 days of pregnancy, AST activity was significantly elevated in GI compared with GII and the control (*P* < 0.001). Additionally, at 21 days of pregnancy, ALT activity was significantly higher in GI than in GII and the control (*P* = 0.003). After two months, ALT activity in GI was significantly lower than that of the control (*P* = 0.009).

The effects of dietary *Azolla* substitution on the lipid profile are also summarized in Table 4. After one month, all lipid parameters in GII—including TC, HDL-C, TG, and VLDL-C—were significantly higher than those in GI and the control (*P* < 0.01), except for LDL-C (*P* = 0.11). Lipid values in GI were also significantly higher than those of the control (*P* < 0.05). After two months, lipid concentrations in GII increased further and remained significantly higher than those of GI and the control (*P* < 0.001). Total cholesterol in GI was also significantly elevated compared with the control (*P* < 0.001). At 21 days of pregnancy, the control group showed significantly lower TC, HDL-C, and LDL-C levels than both substitution groups (*P* < 0.01), whereas LDL-C, TG, and VLDL-C were significantly higher in GII compared with the other treatments (*P* < 0.001).

Concerning kidney function, GI exhibited significantly lower **urea concentrations** than GII and the control after one month and at 21 days of pregnancy (*P* = 0.009 and *P* = 0.007, respectively). In contrast, serum creatinine levels did not differ significantly among groups throughout the experiment (*P* = 0.07).

### Effect of Azolla on immunoglobulin and hormones

The effects of substituting soybean meal protein with dried *Azolla* on serum immunoglobulins are summarized in Table 5. After one month, IgM levels were significantly lower in GII (100% replacement) compared with GI (50% replacement) and the control group (*P* = 0.008). In contrast, IgG levels did not differ significantly at this time point (*P* > 0.05). After two months, IgG concentrations were significantly reduced in GI compared with GII and the control (*P* < 0.001), while IgM levels showed no significant differences among the groups (*P* = 0.06).

**Table 5:** Effect of soybean meal protein substitution with dried Azolla on serum Immunoglobulin G and M levels in female rabbits at different times

	Group	IgG (mg/ml)	IgM (mg/ml)
After a month	Control	183.33 ± 3.86	25.10 ± 0.8
	**G I**	188.33 ± 4.82	24.15 ± 1.39
	**G II**	180.17 ± 2.27	21.00 ± 1.19^a, b^
After two months	Control	231.67 ± 3.74	31.17 ± 1.36
	**G I**	208.00 ± 4.21^a^	30.01 ± 0.40
	**G II**	228.83 ± 5.51^b^	31.05 ± 1.88

Values are presented as means ± SE (*n* = 7). G I; Azolla (50% of soybean protein), G II; Azolla (100% of soybean protein). ^a^ significant difference as compared to control and ^b^ significant differences as compared to G I (*P* < 0.05). IgG is immunoglobulin G and IgM is immunoglobulin M

**Fig. 3:**
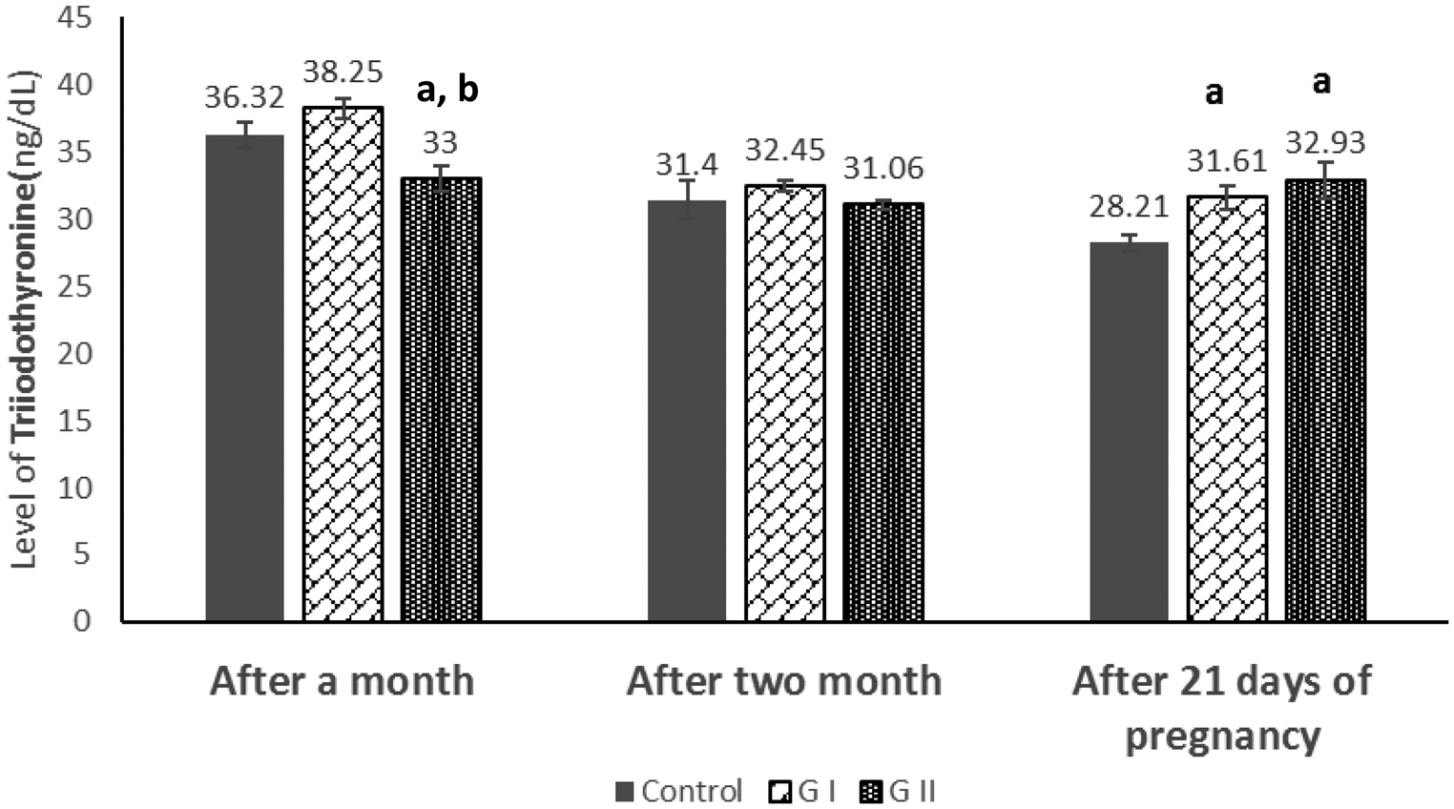
Effect of soybean meal protein substitution with dried Azolla on serum triiodothyronine (T3) hormones in female rabbits at different times. Values are presented as means ± SE (*n* = 7). G I; Azolla (50% of soybean protein), G II; Azolla (100% of soybean protein). ^a^ significant difference as compared to control, and ^b^ significant differences as compared to G I (*P* < 0.05)

**Fig. 4:**
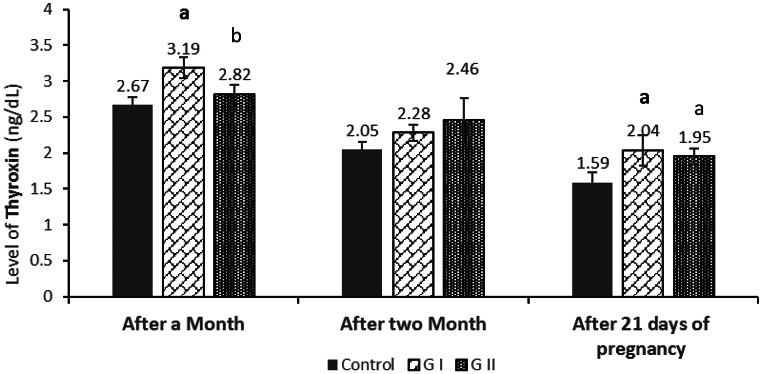
Effect of soybean meal protein substitution levels with dried Azolla on serum Thyroxin (T4) hormones of female rabbits at different times Values are presented as means ± SE (*n* = 7). G I; Azolla (50% of soybean protein), G II; Azolla (100% of soybean protein). ^a^ significant difference as compared to control, and ^b^ significant differences as compared to G I. (*P* < 0.05)

After one month, T3 levels were significantly lower in GII compared with GI and the control (*P* = 0.009; Fig. 3), whereas T4 concentrations were significantly higher in GI than in GII and the control (*P* = 0.004; Fig. 4). After two months, no significant differences in T3 or T4 were observed among the groups (*P* > 0.05). **At** 21 days of pregnancy, both T3 and T4 were significantly lower in the control group compared with GI and GII (*P* = 0.012 for T3; *P* = 0.015 for T4), indicating an effect of dietary Azolla on thyroid hormone levels during early gestation.

**Fig. 5:**
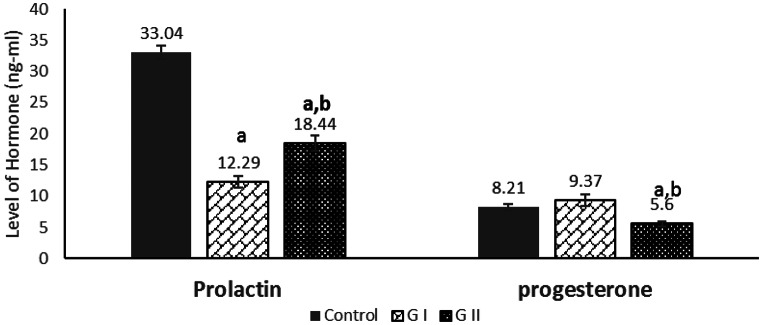
Effect of soybean meal protein substitution with dried Azolla on serum prolactin and progesterone levels of female rabbits after 21 days of pregnancy. Values are presented as means ± SE (*n* = 7). G I; Azolla (50% of soybean protein), G II; Azolla (100% of soybean protein). ^a^ significant difference as compared to control, and ^b^ significant differences as compared to G I (*P* < 0.05)

**Fig. 6:**
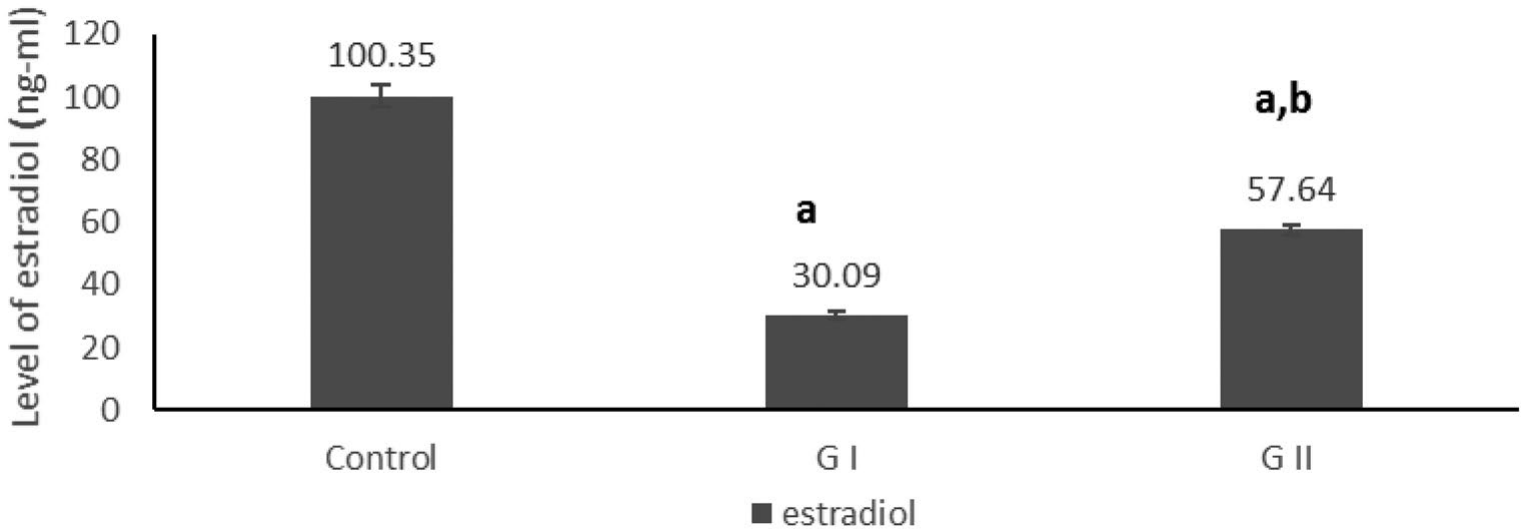
Effect of soybean meal protein substitution levels with dried Azolla on serum estradiol levels of female rabbits after 21 days of pregnancy. Values are presented as means ± SE (*n* = 7). G I; Azolla (50% of soybean protein), G II; Azolla (100% of soybean protein). ^a^ significant difference compared to control, and b significant differences compared to G I (*P* < 0.05)

The effects of dried Azolla substitution on reproductive hormones (progesterone, prolactin, and estradiol) are presented in Figs. 5 and 6. Prolactin was significantly decreased in GI compared with the control (*P* = 0.021). In GII, both prolactin and progesterone were significantly lower than in the control (*P* = 0.008 and *P* = 0.012, respectively). Estradiol levels were significantly reduced in GI compared with GII and the control (*P* = 0.018). Additionally, prolactin in GII was significantly lower than in the control (*P* = 0.009). These findings indicate that dietary replacement of soybean meal protein with dried Azolla modulates reproductive hormone profiles, with more pronounced effects at 100% replacement.

### Effect of Azolla on oxidative stress/antioxidant status

The effects of substituting dietary soybean meal protein with dried *Azolla* on oxidative stress and antioxidant parameters including MDA, SOD, CAT, GSH, and TAC are summarized in Table 6.

**Table 6:** Effect of soybean meal protein substitution with dried Azolla on serum of oxidative and antioxidant parameters of female rabbits at different times

	Groups	MAD (nmol/ml)	SOD (U/ml)	CAT (ng/ml)	GSH (ng/ml)	TAC (ng/ml)
After1 Month	Control	3.21 ± 0.17	98.30 ± 2.09	3.86 ± 0.11	95.08 ± 2.09	2.36 ± 0.09
	G I	6.95 ± 0.18^a^	49.35 ± 1.76^a^	1.34 ± 0.16^a^	56.10 ± 2.85^a^	1.09 ± 0.14 ^a^
	G II	1.34 ± 0.19^a, b^	161.01 ± 4.38^a, b^	7.07 ± 0.13 ^a, b^	170.64 ± 3.97^a, b^	4.16 ± 0.16^a, b^
After2 Month	Control	4.10 ± 0.20	114.34 ± 3.82	3.55 ± 0.18	114.96 ± 3.70	1.90 ± 0.24
	G I	13.29 ± 0.40^a^	26.88 ± 1.83^a, b^	0.56 ± 0.03^a^	23.33 ± 1.24^a^	0.51 ± 0.04^a^
	G II	7.21 ± 0.27^a, b^	77.89 ± 2.29^a, b^	1.32 ± 0.17^a, b^	76.50 ± 3.99^a, b^	0.84 ± 0.04^a, b^

Values are presented as means ± SE (*n* = 7). G I; Azolla (50% of soybean protein), G II; Azolla (100% of soybean protein). Lipid peroxidation (MDA), superoxide dismutase (SOD), catalase (CAT), glutathione (GSH), and total antioxidant capacity (TAC). ^a^ significant difference compared to control, and ^b^ significant differences compared to G I (*P* < 0.05)

After one month, MDA levels were significantly higher in GI (50% replacement) compared with GII (100% replacement) and the control group (*P* = 0.011). Interestingly, MDA levels in the control group were also significantly higher than in GII at this time point (*P* = 0.023).

In contrast, antioxidant enzyme activities including SOD, CAT, GSH, and TAC were significantly elevated in GII compared with GI and the control (*P* < 0.01 for all parameters). Additionally, the control group exhibited significantly higher antioxidant levels than GI (*P* < 0.05), indicating a dose-dependent improvement in antioxidant status with higher Azolla inclusion.

After two months, MDA concentrations were significantly lower in the control group compared with GII (*P* = 0.015), while levels in GI remained elevated. Conversely, antioxidant parameters (SOD, CAT, GSH, and TAC) were significantly higher in the control group than in the experimental groups (*P* < 0.01). Within the experimental groups, GII maintained higher antioxidant levels than GI (*P* < 0.05), demonstrating that partial or complete substitution of soybean meal protein with dried Azolla can enhance the antioxidant defense system over time.

### Effect of Azolla on carcass traits of growing rabbits

The impact of replacing soybean meal with dried *A. pinnata* on carcass characteristics of female rabbits is presented in Table 7. Live body weights did not differ significantly among the control, GI (50% replacement), and GII (100% replacement) groups (*P* > 0.05), indicating that overall growth was not compromised by dietary Azolla inclusion.

**Table 7:** Effect of soybean meal protein substitution with dried Azolla on carcass traits of growing rabbits (% from LBW)

Azolla (% of soybean protein)				
Traits	Control	G Ι	G Π	
Live weight (g)	2631.66 ± 53.74	2506.66 ± 55.68	2640.00 ± 50.32	
Heart (%)	0.40 ± 0.03	0.31 ± 0.06	0.29 ± 0.05	
Liver (%)	2.70 ± 0.15	2.41 ± 0.17	2.80 ± 0.14	
Front quarter (%)	9.07 ± 0.35	8.75 ± 0.37	8.76 ± 0.31	
Back quarter (%)	21.83 ± 0.47	21.22 ± 50	20.09 ± 0.45	
Lumber (%)	12.34 ± 0.59	12.10 ± 0.61	11.91 ± 0.52	
Lung (%)	0.89 ± 0.05	0.96 ± 0.04	0.84 ± 0.04	
Kidney (%)	0.60 ± 0.06	0.67 ± 0.03	0.65 ± 0.05	

Values are presented as means ± SE (*n* = 7). G I; Azolla (50% of soybean protein), G II; Azolla (100% of soybean protein)

Minor variations were observed in organ and carcass component percentages. Heart weight showed a slight reduction in Azolla-fed groups, while liver percentage exhibited minor fluctuations; however, these differences were not statistically significant (*P* > 0.05). Front quarter percentages were consistent across all groups, whereas back quarter and lumbar percentages were slightly lower in Azolla-fed rabbits, particularly in GI, suggesting a negligible effect on regional muscle deposition. Lung and kidney percentages remained stable among all groups (*P* > 0.05).

Overall, substituting soybean meal with dried *A. pinnata* did not significantly affect carcass traits, demonstrating that Azolla can serve as an effective protein alternative without compromising carcass quality.

## Discussion

The current study evaluated the viability of replacing soybean meal with sun‑dried *A*. *pinnata* in diets for New Zealand White (NZW) female rabbits, analyzing impacts on growth, productivity, and physiological health. This research is timely given the global push toward sustainable, cost‑effective feed solutions.

Before incorporating any ingredient into livestock feed, it’s essential to understand both its nutritional content and availability. In the current study, the crude protein (CP) content of Azolla was found to be 19.4%. This value is higher than those reported by El-Fadel et al. (2020), though some recent studies report even higher CP levels (Normuhammedova and Rajamurodov 2025).

According to Sadeghi (2013), variations in Azolla’s nutrient composition are largely due to differences in how various strains respond to environmental conditions such as temperature, light intensity, and soil nutrients—factors that influence both growth and nutritional makeup. Alalade and Iyay (2006) also noted that the presence of epiphytic algae contamination can significantly affect the amino acid profile, further influencing the final nutritional analysis.

Partial replacement (50% protein substitution) supported growth performance and physiological balance, whereas full replacement (GII) led to growth retardation despite lower birth mortality and some reproductive benefits—mirroring research that reports moderate inclusion as beneficial while high levels may introduce anti-nutritional effects (Samad et al. 2020; Abdelatty et al. 2021).

In terms of serum proteins, the elevated total protein and globulin levels observed in GII after one month may be attributed to Azolla’s rich amino acid profile. However, the subsequent decline in these parameters after two months suggests potential long-term limitations in the bioavailability of Azolla’s nutrients when used as the sole protein source, as supported by previous findings (Méndez-Martínez et al. 2019; Abdelatty et al. 2021). Liver enzyme alterations may reflect adaptive metabolic responses: moderate Azolla promotes enzyme efficiency, but high levels may elicit metabolic stress, consistent with studies in similar species Similarly, the stable creatinine levels across all groups suggest that kidney function remained largely unaffected, while urea levels were lower in GI, indicating efficient protein metabolism at moderate Azolla levels (Leja et al. 2007; Nasir et al. 2022).

Although the control diet had higher crude protein content, the superior digestibility, amino acid profile, and bioactive constituents of dried *A. pinnata* likely contributed to improved growth, antioxidant capacity, and reproductive parameters in the supplemented groups, demonstrating that protein quality and functional nutrients are as important as total protein content.

Azolla inclusion led to significant increases in total cholesterol, triglycerides, and LDL-c, particularly in the GII group. While Azolla is rich in bioactive compounds, high fiber, and anti-nutritional components may have interfered with lipid metabolism, aligning with previous observations in rabbits and poultry (Kathirvelan et al. 2015; Ogbuewu et al. 2017). These findings suggest that excessive Azolla inclusion could disrupt lipid homeostasis.

Immune markers (IgM, IgG) varied with dosage—partial supplementation maintained immune competence, while high Azolla suppressed Ig levels. Previous studies noted that moderate levels of Azolla enhance immune responses, while higher levels may suppress immunity due to potential anti-nutritional factors (Mishra et al. 2016; Ismail et al. 2023). *A. pinnata*, in its green state, contains beta-carotene and antioxidant vitamin A, which possess antioxidant activity that may contribute to enhanced immune health (El Naggar and El-Mesery 2022).

The results of this study indicate that Azolla supplements can affect T3 and T4 thyroid hormones levels reproductive hormones in rabbits, with effects varying depending on the duration of exposure to Azolla and the physiological state of the animals. Maintaining thyroid hormone levels within the normal physiological range is crucial for regulating basal metabolic rate and ensuring the proper function of various essential bodily processes (Kim 2008). T3 and T4 functions as thermoregulatory in animals and are critical for metabolic activities associated with nutrition and environmental adaptation. These hormones regulate key processes such as energy balance, protein metabolism, and thermoregulation as indicated by Bhatt et al. 2022.

The improved antioxidant status (SOD, CAT, GSH, TAC) observed in GII after one month may be linked to Azolla’s rich antioxidant content, including vitamins and bioactive compounds (Chichilichi et al. 2015). However, prolonged inclusion resulted in fluctuating MDA levels, indicating oxidative stress at higher Azolla levels. This pattern is consistent with other studies emphasizing the dose-dependent antioxidant benefits of Azolla (Kamel and Hamed 2021; Punyatong et al. 2024).

The present study examined the effects of dried Azolla in the diet on oxidative stress and antioxidant markers, including MDA, SOD, CAT, GSH, and TAC. The varying changes in MDA levels suggest that Azolla’s impact on oxidative stress is not straightforward, potentially influenced by both the amount included in the diet and the duration of exposure. MDA, a key product of lipid peroxidation, is an important indicator of oxidative damage, as noted by Kaya and Kaptaner 2016. The observed increase in SOD, CAT, GSH, and TAC in the GII group after one month of feeding may reflect a compensatory mechanism to mitigate oxidative stress. Azolla could have stimulated the upregulation of antioxidant enzymes and molecules.

The presence of iron and copper in Azolla likely contributed to the increased CAT activity in broiler erythrocytes. CAT, a heme enzyme, relies on these metals to effectively convert hydrogen peroxide into water, following the activity of superoxide dismutase, as reported by Chichilichi et al. 2015. By two months, a reduction in antioxidant levels in the GII group, in combination with changes in other groups, suggests a dynamic interaction between Azolla supplementation, time-related factors, and the modulation of antioxidant defenses in rabbits. Dietary components can significantly influence the activity of antioxidant enzymes and the concentration of antioxidant molecules, as discussed by Virgili and Marino 2008.

Maintaining the relative proportions of major carcass quarters (Front Quarter, Hind Quarter, Lumbar Region) suggests that the protein quality and amino acid profile provided by Azolla are adequate, even if they differ from those of soybean meal (Vahedi et al. 2021). The stability of these relative organ weights suggests that Azolla inclusion did not impose undue metabolic stress or lead to organ hypertrophy or atrophy. This is crucial for animal health and welfare, as significant changes in organ size can reflect physiological dysfunction reported by Hamid et al. (2026).

## Conclusion

Azolla is a protein-rich floating fern with essential nutrients but low carbohydrates and lipids. It is valuable for animal feed, biofertilization, and bioremediation. Overall, the study highlights the potential of *A. pinnata* as a sustainable, cost-effective partial replacement for soybean meal protein in rabbit diets. While 50% replacement supports growth, productivity, biochemical balance, and antioxidant status without adverse effects, complete replacement negatively impacts growth, lipid metabolism, and certain hormonal parameters. These findings suggest that moderate inclusion levels of Azolla optimize its nutritional benefits while avoiding the drawbacks associated with high inclusion rates. Future research should focus on processing techniques to reduce anti-nutritional factors in Azolla, improving its viability as a complete protein substitute.

## Data Availability

The data supporting the findings of this study are available from the corresponding author upon reasonable request.

## References

[CR1] Abdelatty 1-AM, Mandouh MI, Mousa MR, Mansour HA, Ford H, Shaheed IB, Elolimy AA, Prince A, El-Sawy MA, AbuBakr HO, Bionaz M (2021) Sun-dried Azolla leaf meal at 10% dietary inclusion improved growth, meat quality, and increased skeletal muscle Ribosomal protein S6 kinase β1 abundance in growing rabbits. Animal 15(10):100348 10.1016/j.animal.2021.100348 34543996

[CR2] Abdelhadi SH, El-Wahab A, Walaa M (2022) Influence of emulsified and nano-emulsified essential oils blend on performance and meat characteristics of weaned mountain rabbits. J Anim Poult Prod 13(3):43–50

[CR3] Adzman N, Goh SJ, Johari A, Alam MZ, Kamaruddin MJ (2022) Preliminary study on Azolla cultivation and characterization for sustainable biomass source. In Journal of Physics: Conference Series, 2259(1):012018. IOP Publishing

[CR4] Alalade, Iyayi EA (2006) Chemical composition and the feeding value of *Azolla pinnata* meal for egg-type chicks. Int J Poult Sci 2:137–141

[CR5] Allen DM (1974) The relationship between variable selection and data agumentation and a method for prediction. Technometrics 16(1):125–127

[CR6] Alshelmani MI, Abdalla EA, Kaka U, Basit MA (2021) Nontraditional feedstuffs as an alternative in poultry feed. In Advances in poultry nutrition research. IntechOpen

[CR7] Alshelmani MI, El-Safty SA, Kairalla MA, Humam AM (2024) Enzymes in poultry feed. Feed Addit-Recent Trends Anim Nutr

[CR8] Anitha KC, Rajeshwari. YB, Prabhu TM, Devarnvadagi AS, Rohith KJ, Shilpa J, Shree (2016) Carcass and Meat Quality Traits of Broiler Rabbits When Supplement with Azolla. J Experimental Zool India 19(1):417–420

[CR9] AOAC (2005) Association of Official Analytical Chemists, Official Methods of Analysis. 18th Edition., Maryland, USA

[CR10] Arora A, Nandal P, Chaudhary A (2022) Critical evaluation of novel applications of aquatic weed Azolla as a sustainable feedstock for deriving bioenergy and feed supplement. Environ Reviews 31(2):195–205

[CR11] Bartel H, Bohmer M, Heierli C (1972) Serum creatinine determination without protein precipitation. Clin Chem Acta, (37):193–197 10.1016/0009-8981(72)90432-9 5022083

[CR12] Bhatt N, Chandra R, Chikkagoudara KP, Kandpal D, Misra DB, Tyagi N (2022) Effects of replacing the protein content of *Azolla Pinnata* with concentrate on physiological and blood profiles changes in Sahiwal calves. Turkish J Veterinary Anim Sci 46(5):724–733

[CR13] Chichilichi B, Mohanty GP, Mishra SK, Pradhan CR, Behura NC, Das A, Behera K (2015) Effect of partial supplementation of sun-dried Azolla as a protein source on the immunity and antioxidant status of commercial broilers. Veterinary world 8(9):1126 27047208 10.14202/vetworld.2015.1126-1130 PMC4774782

[CR14] Doumas BT, Watson WA, Biggs HG (1971) Albumin standards and the measurement of serum albumin with bromcresol green. Clin Chim Acta 31(1):87–96 5544065 10.1016/0009-8981(71)90365-2

[CR15] El Naggar S, El-Mesery HS (2022) *Azolla pinnata* as unconventional feeds for ruminant feeding. Bull Natl Res Centre 46(1):66

[CR16] El-Fadel A, Hassanein HAM, El-Sanafawy HA (2020) Effect of partial replacement of protein sun flower meal by Azolla meal as source of protein on productive performance of growing lambs. J Anim Poult Prod 11:149–153

[CR17] El-Sabrout K, Khalifah A, Ciani F (2023) Current applications and trends in rabbit nutraceuticals. Agriculture 13(7):1424

[CR18] Fassati P, Prencipe L (1982) Triglyceride enzymatic colorimetric method. J Clin Chem 28(3):77–72

[CR19] Food and Agriculture Organization of the United Nations (FAO) (2023) The state of food security and nutrition in the world 2023

[CR20] Henry FM (1964) Physical education: An academic discipline. J Health Phys Educ Recreation 35(7):32–69

[CR21] Herath BMMD, Karunarathna SC, Ishaq M, Wariss HM, Yapa PN (2023) Azolla as the multifunctional fern in organic agriculture: prospects and challenges: a review article. Int J Agricultural Technol 19(1):63–82

[CR22] Ismail MA, Attia AI, Mohamed LA, Alagawany M (2023) Effect of varying dietary Azolla levels on growth, carcass characteristics, blood biochemical parameters, and digestive enzymes of growing Egyptian geese. Animal Biotechnol 34(8):3441–3448 10.1080/10495398.2022.2154224 PMC13353326 36520017

[CR23] Kairalla MA, Alshelmani MI, Muftah IM, Aburas AA (2025) Effect of inclusion corn distillers’ dried grains with soluble on performance, carcass and meat quality on broiler chicken. Zanco J Pure Appl Sci 37(4):70–77

[CR24] Kamel ER, Hamed E (2021) Effect of dried azolla on growth performance, hematological, biochemical, antioxidant parameters, and economic efficiency of broiler chickens. Adv Anim Veterinary Sci 9(11):1886–1894

[CR25] Kathirvelan C, Banupriya S, Purushothaman MR (2015) Azolla an alternate and sustainable feed for livestock. Int J Sci Environ Technol 4(4):1153–1157

[CR26] Kaya Ö, Kaptaner B (2016) Antioxidant defense system parameters in isolated fish hepatocytes exposed to bisphenol A—effect of vitamin C. Acta Biol Hung 67:225–235 27630046 10.1556/018.67.2016.3.1

[CR27] Khan FU, Ullah R, Kinkpe L, Hassan SU, Ahamba IS, Goswami N, Shuaib M (2024) Substitution of Soybean meal with *Azolla pinnata* meal improves gut histomorphology and growth performance in commercial broilers. Brazilian J Poult Sci 26:eRBCA–2024

[CR28] Kim B (2008) Thyroid hormone as a determinant of energy expenditure and the basal metabolic rate. Thyroid 18(2):141–144 18279014 10.1089/thy.2007.0266

[CR29] Kollah B, Patra AK, Mohanty SR (2016) Aquatic microphylla Azolla: a perspective paradigm for sustainable agriculture, environment, and global climate change. Environ Sci Pollut Res 23:4358–4369 10.1007/s11356-015-5857-9 26697861

[CR30] Korsa G, Alemu D, Ayele A (2024) Azolla plant production and their potential applications. Int J Agron 20241:1716440

[CR31] Kouchakinejad R, Lotfi Z, Golzary A (2024) Exploring Azolla as a sustainable feedstock for eco-friendly bioplastics: a review. Heliyon, 10(20) 10.1016/j.heliyon.2024.e39252 PMC11620271 39640731

[CR32] Lee WY, Nieman TA (1996) Effect of organic solvent on tris (2, 2′-bipyridyl) ruthenium (III) chemiluminescent reactions in flowing streams. Anal Chim Acta 334(1–2):183–191

[CR33] Leja M, Mareczek A, Wyzgolik G, Klepacz-Baniak J, Czekon´ska K (2007) Antioxidative properties of bee pollen in selected plant species. Food Chem 100:237–240

[CR34] Lopez MM (1977) Vertical and temporal distribution of phytoplankton populations of Lake Tahoe, California-Nevada. University of California, Davis

[CR35] Love DC, Allison EH, Asche F, Belton B, Cottrell RS, Froehlich HE, Zhang W (2021) Emerging COVID-19 impacts, responses, and lessons for building resilience in the seafood system. Global Food Secur 28:100494 10.1016/j.gfs.2021.100494 PMC8417121 34513582

[CR36] Méndez-Martínez Y, Ramírez JL, Álvarez AR, Leyva L, Pérez Y (2019) Partial substitution of commercial concentrate for *Azolla filiculoides* meal in the productive response of Oryctolagus cuniculus. Cuban J Agricultural Sci 53(2):149–159

[CR37] Mishra DB, Roy D, Kumar V, Bhattacharyya A, Kumar M, Kushwaha R, Vaswani S (2016) Effect of feeding different levels of *Azolla pinnata* on blood biochemicals, hematology, and immunocompetence traits of Chabro chicken. Veterinary World 9(2):192 27051207 10.14202/vetworld.2015.192-198 PMC4819371

[CR38] Mottet A, Tempio G (2017) Global poultry production: current state and future outlook, and challenges.World’s. Poult Sci J 73:245–256

[CR39] Nasir NANM, Kamaruddin SA, Zakarya IA, Islam AKMA (2022) Sustainable alternative animal feeds: Recent advances and future perspective of using Azolla as animal feed in livestock, poultry, and fish nutrition. Sustainable Chem Pharm 25:100581

[CR40] National Research Council (1977) The Nutrient Requirements of Rabbits. National Academy of Sciences, Washington, DC

[CR41] Nayel U, Baraghit G, Elaref M, Abdelhakeem MA, Saddick E (2024) Impact of Azolla plant on digestibility, nutritive value and rumen fermentation in barki sheep diets. Menoufia J Anim Poult Fish Prod 8(2):11–20

[CR42] Normuhammedova F, Rajamurodov Z (2025) The Nutritional Value of Azolla caroliniana Wild as Animal Feed. Am J Plant Sci 16(3):369–377

[CR43] Odero-Waitituh JA (2021) Reduction of anti-nutritive compounds in ground mature Prosopis juliflora pods in rabbit diets using fermentation technology. Thesis

[CR44] Ogbuewu IP, Emenalom OO, Okoli IC (2017) Alternative feedstuffs and their effects on blood chemistry and haematology of rabbits and chickens: a review. Comp Clin Pathol 26:277–286

[CR45] Patton FG, Grouch SR (1977) Colorimetric determination of urea. Anal Chem 49:464–468

[CR46] Punyatong M, Kanjak P, Tapingkae W, Lumsangkul C, Moonmanee T, Van Doan H, Khamtavee P (2024) Effect of fresh azolla (*Azolla pinnata*) feed replacement on growth performance, carcass quality, and oxidative stress in Thai native crossbred chicken. Veterinary Integr Sci 22(3):1019–1028

[CR47] Rashad S (2021) An overview on the aquatic fern Azolla spp. as a sustainable source of nutrients and bioactive compounds with resourceful applications. Egypt J Aquat Biology Fisheries 25(1):775–782

[CR48] Reitman S, Frankel S (1957) A colorimetric method for the determination of serum glutamic oxalacetic and glutamic pyruvic transaminases. Am J Clin Pathol 28(1):56–63 13458125 10.1093/ajcp/28.1.56

[CR49] Sadeghi R, Zarkami R, Sabetraftar K, Van Damme P (2013) A review of some ecological factors affecting the growth of Azolla spp. Casp J Environ Sci 11(1):65

[CR50] Samad FA, Idris LH, Hassim A, Goh H, Y. M., Loh TC (2020) Effects of Azolla spp. as feed ingredient on the growth performance and nutrient digestibility of broiler chicken. J Anim Physiol Anim Nutr 104(6):1704–1711 10.1111/jpn.13345 32200580

[CR51] Siddiqui SA, Adli DN, Nugraha WS, Yudhistira B, Lavrentev FV, Shityakov S, … Ibrahim SA (2024) Social, ethical, environmental, economic and technological aspects of rabbit meat production-A critical review. Heliyon, 10(8) 10.1016/j.heliyon.2024.e29635 PMC11063435 38699749

[CR52] Vahedi V, Hedayat-Evrigh N, Holman BW, Ponnampalam EN (2021) Supplementation of macro algae (*Azolla pinnata*) in a finishing ration alters feed efficiency, blood parameters, carcass traits and meat sensory properties in lambs. Small Ruminant Res 203:106498

[CR53] Van Dijk M, Morley T, Rau ML, Saghai Y (2021) A meta-analysis of projected global food demand and population at risk of hunger for the period 2010–2050. Nat food 2(7):494–501 37117684 10.1038/s43016-021-00322-9

[CR54] Virgili F, Marino M (2008) Regulation of cellular signals from nutritional molecules: a specific role for phytochemicals, beyond antioxidant activity. Free Radic Biol Med 45(9):1205–1216 18762244 10.1016/j.freeradbiomed.2008.08.001

[CR55] Hamid MMA, Tharwat M, Ebeid TA, Alshanbari FA (2026) Nutritional disorders and metabolic adaptations in Dromedary camels: insights into foregut fermentation and mineral balance. Animals 16(4):689 41751150 10.3390/ani16040689 PMC12937329

[CR56] Lukefahr S, Hohenboken WD, Cheeke PR, Patton NM, Kennick WH (1982) Carcass and meat characteristics of Flemish Giant and New Zealand White purebred and terminal-cross rabbits. J Anim Sci 54(6):1169–1174

